# The Unfolded Protein Response: A Key Player in Zika Virus-Associated Congenital Microcephaly

**DOI:** 10.3389/fncel.2019.00094

**Published:** 2019-03-26

**Authors:** Christian Alfano, Ivan Gladwyn-Ng, Thérèse Couderc, Marc Lecuit, Laurent Nguyen

**Affiliations:** ^1^GIGA-Stem Cells, Interdisciplinary Cluster for Applied Genoproteomics (GIGA-R), University of Liège, Liège, Belgium; ^2^Institut Pasteur, Biology of Infection Unit, Paris, France; ^3^INSERM U1117, Biologie des Infections, Paris, France; ^4^Paris Descartes University, Division of Infectious Diseases and Tropical Medicine, Necker-Enfants Malades Hospital, Institut Imagine, Sorbonne Paris Cité, Paris, France

**Keywords:** unfolded protein response, ER stress, cortical progenitors, cerebral cortex, microcephaly, Zika virus

## Abstract

Zika virus (ZIKV) is a mosquito-borne virus that belongs to the *Flaviviridae* family, together with dengue, yellow fever, and West Nile viruses. In the wake of its emergence in the French Polynesia and in the Americas, ZIKV has been shown to cause congenital microcephaly. It is the first arbovirus which has been proven to be teratogenic and sexually transmissible. Confronted with this major public health challenge, the scientific and medical communities teamed up to precisely characterize the clinical features of congenital ZIKV syndrome and its underlying pathophysiological mechanisms. This review focuses on the critical impact of the unfolded protein response (UPR) on ZIKV-associated congenital microcephaly. ZIKV infection of cortical neuron progenitors leads to high endoplasmic reticulum (ER) stress. This results in both the stalling of indirect neurogenesis, and UPR-dependent neuronal apoptotic death, and leads to cortical microcephaly. In line with these results, the administration of molecules inhibiting UPR prevents ZIKV-induced cortical microcephaly. The discovery of the link between ZIKV infection and UPR activation has a broader relevance, since this pathway plays a crucial role in many distinct cellular processes and its induction by ZIKV may account for several reported ZIKV-associated defects.

## Overview of the Zika Epidemics

Zika virus (ZIKV) was first isolated in 1947 from the Zika forest in Uganda, East Africa (Dick et al., 1952), and identified as a member of the *Flaviviridae* genus (as yellow fever, West Nile, Japanese encephalitis, and dengue viruses). However, it remained until recently largely unknown, with fewer than 20 confirmed human infections reported over the 60 years prior to 2007 (Musso and Gubler, 2016). It has likely, however, circulated undetected during this time. This may result from (i) clinical or serological misdiagnosis with life threatening arboviral infections (such as dengue), (ii) the absence of known severe outcome favoring silent transmission, and (iii) occurrence in places where epidemiological and medical infrastructure are scarce and which are overwhelmed by more severe medical threats (Musso and Gubler, 2016). After ZIKV emergence in Western Pacific in Yap Island in 2007 (Lanciotti et al., 2008; Duffy et al., 2009), and in French Polynesia, South Pacific in 2013–2014 (Oehler et al., 2014), ZIKV reached the Americas, particularly Brazil in 2015, with 440,000–1,300,000 reported cases (Bogoch et al., 2016).

In Brazil, soon after the emergence of ZIKV, microcephalic newborns from ZIKV-infected mothers were reported (Coelho and Crovella, 2017; Saiz et al., 2017), as well as children with postnatal neural and non-neural disorders following congenital ZIKV infection (McCarthy, 2016; Moore et al., 2017; Mavigner et al., 2018). This led to the identification of ZIKV as a teratogenic agent (Baud et al., 2017; Schaub et al., 2017b). Additionally, ZIKV has been shown to be transmitted sexually (Matusali et al., 2018; Sakkas et al., 2018) and more likely transmitted from men to women than the converse (Counotte et al., 2018), with an increased risk of male infertility (Caswell and Manavi, 2018). Furthermore, ZIKV infection of adults has been causally linked to Guillain-Barré syndrome (GBS) (Cao-Lormeau et al., 2016). The emergence of ZIKV in the Pacific and the Americas, and the identification of severe neurological complications led the declaration of ZIKA as a Public Health Emergency of International Concern (PHEIC) (WHO, 2016^1^). The concurrence of this epidemic with the 2016 Summer Olympic Games in Rio de Janeiro further crystallized the global scrutiny on this virus.

## Open Questions on Zika Pathogenesis

Toward understanding the underlying mechanisms and pathogenesis of ZIKV infections, molecular studies were performed with cutting edge tools, such as human induced pluripotent stem cells (hiPSCs), as well as human cell-derived neurospheres and organoids (Cugola et al., 2016; Tang et al., 2016; Garcez et al., 2017; Himmelsbach and Hildt, 2018). These tools shed some light on the ZIKV pathophysiological mechanisms on human-derived samples, and these experiments were further corroborated by studies performed *in vivo* in animals and in immortalized cell lines. Indeed, the initial reports from different groups unveiled a strong tropism of ZIKV for neuronal precursors of the cerebral cortex (Cugola et al., 2016; Li et al., 2016; Tang et al., 2016; Gladwyn-Ng et al., 2018). In combination, these findings supported that ZIKV impairs cell cycle progression, and triggers a cascade of apoptotic and autophagic cell deaths that ultimately lead to microcephaly. A recent study reported that ZIKV-induced transcriptional effects in human neural progenitors that were similar to those elicited by numerous genetic mutations underlying severe microcephaly in mice (Ghouzzi et al., 2016; Zhang et al., 2016). The massive cell loss observed in ZIKV-infected brains is likely to account for cortical lesions with impaired neuronal connectivity, able to induce postnatal neurological dysfunctions such as epilepsy, secondary microcephaly, cognitive defects, that constitute frequent outcomes of congenital ZIKV syndrome (CZS) (Franca et al., 2018; Subissi et al., 2018; van der Linden et al., 2018). These reports supported a causal link between ZIKV infection and the neurological impairments observed *in utero*-infected infants.

## Zika Infection Induces Endoplasmic Reticulum Stress and Activation of the Unfolded Protein Response

**FIGURE 1:**
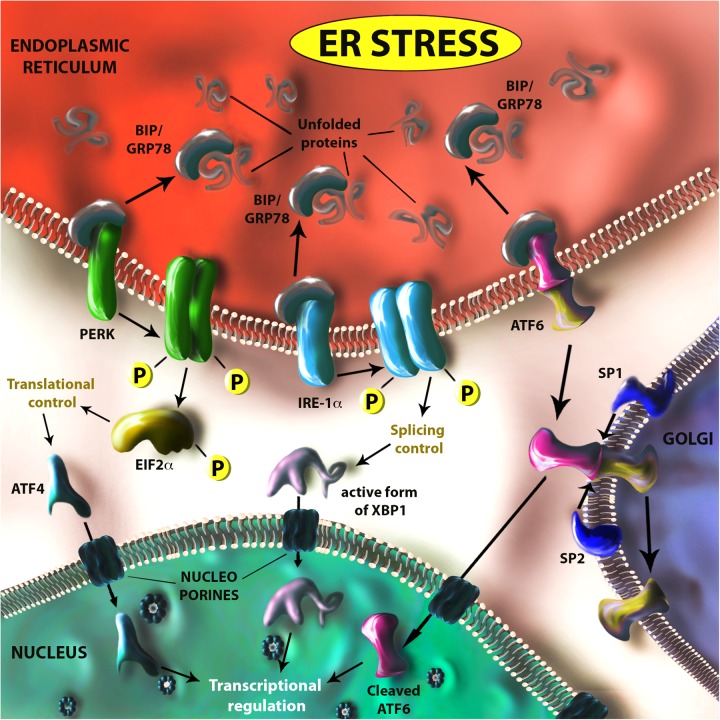
Schematic representation of the unfolded protein response (UPR). In case of ER stress (due to viral infection, metabolic impairments, inflammation, cancer, hypoxia, etc.) translated proteins improperly folded are recognized by the endoplasmic reticulum (ER) chaperon GRP78. This protein is usually bound to three ER receptors (PERK, IRE-1α, and ATF6), which are maintained in an inactive form. When GRP78 binds to unfolded proteins it releases these three receptors, which are activated either through dimerization and auto-phosphorilation (PERK and IRE-1α) or by translocation and cleavage (through SP1 and SP2 proteases) on Golgi apparatus membrane (ATF6). The PERK-controlled pathway is mainly deputed to the control of translational activity in the ER through EIF2α phosphorilation, but has also an active role in transcriptional control by regulating ATF4 translation. IRE-1α and ATF6, together with ATF4, have an important role in the transcriptional control of genes involved in UPR regulated processes such as apoptosis, autophagy, inflammation, and angiogenesis, etc. IRE-1α regulates XBP1 activity by modulating its RNA-splicing.

Zika virus, as a single stranded RNA virus replicates in the endoplasmic reticulum (ER) of host cells, which was recently reported to be targeted by the interferon (IFN) response (Richardson et al., 2018). ZIKV infection causes ultrastructural changes of the ER and ER-derived structures in nerve cells in mouse brains infected after birth *in vivo* and hepatocarcinoma cells *in vitro* (Bell et al., 1971; Cortese et al., 2017). In addition, ZIKV induces massive vacuolization (which are derived from the ER) followed by cell death in human astrocytes and others such as primary skin fibroblasts and epithelial cells (Monel et al., 2017). ZIKV infection also perturbs ER-related pathways that are connected to other relevant pathways such as apoptosis and innate immunity (Blazquez et al., 2014). Stressors of ER physiological functions have led to the selection of an evolutionarily conserved cell response that comprises multiple effector pathways, collectively known as the unfolded protein response (UPR). Although the UPR has been described as a homeostatic compensation for cell stress, it can potentially trigger cell death after severe or prolonged ER dysfunction (reviewed in Xu et al., 2005). UPR is modulated by three critical ER-membrane bound receptors: PKR-like endoplasmic reticulum kinase (PERK), Inositol-requiring enzyme 1 (IRE1α) and activating transcription factor 6 (ATF6) (see Figure 1; Godin et al., 2016). Collectively, these pathways constitute a finely tuned sensor of ER activity during altered protein translation and folding (resulting for example from oxidative stress, hypoxia, infections), which mainly act by (i) reducing the workload of the ER through the PERK-mediated pathway, which mainly slows down ribosomal activity and (ii) modulating the transcription of several effectors regulating different cellular events through IRE1α- and ATF6-mediated pathways (such as cell cycle to cell survival, from autophagy to cytoskeletal remodeling). The pharmacological modulation of UPR has been explored as a potential therapeutic strategy against a diverse gamut of pathologies such as cancer, neurodegenerative and metabolic diseases (e.g., diabetes) as well as age-related dysfunctions (e.g., age-related retinopathies) (Scheper and Hoozemans, 2015; Galmiche et al., 2017). Interestingly, many of the cellular dysfunctions observed in ZIKV-infected cells (such as deregulation of homeostasis and cell cycle) would fit with the induction of ER stress and the ensuing UPR.

## A Crucial Link Between UPR and Neurogenesis

**FIGURE 2:**
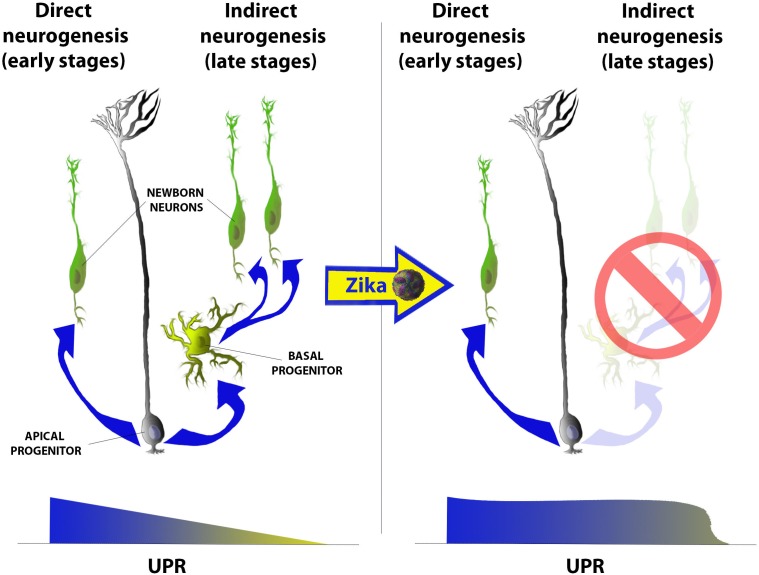
Schematic representation of the gradual shift from direct (left) to indirect (right) neurogenesis and of its imbalance after Zika infection in mammalian cerebral cortices. Briefly, at early stages of corticogenesis (from E10.5 to E13.5 in mice) apical progenitors (AP) populating the ventricular zone (VZ) of the rostral neural tube start producing first neurons, which will give rise to the primordial cortical plate (CP). At these stages the level of UPR is higher then later stages and gradually decreases as development proceeds. From E13.5 onward (in mice) AP start producing at least other two progenitor populations: basal progenitors and outer radial glia (not in the scheme) and, as UPR decreases, this neurogenic mode (called indirect) becomes gradually predominant. Indirect neurogenesis is only present in mammals (among all vertebrates) and mainly contributes to the amplification of the neuronal output during corticogenesis. Upon Zika infection, instead, UPR levels remain high also at later stages of development and the predominant mode of neurogenesis remains the direct one leading to a decrease in the total neuronal output and, hence, to a microcephalic cortex.

The UPR pathway was first shown to be fundamental for temporal regulation neurogenesis in the mammalian cerebral cortex during development (Laguesse et al., 2015a). Neural progenitors [also called apical progenitors (AP)], located at the luminal surface of the rostral neural tube (telencephalon), predominately generate neurons that migrate basally during the earlier stages of cortical development. This process is termed direct neurogenesis and gives rise to the primordial cerebral cortex (Figure 2, left). Toward later stages of cortical development, AP sequentially give rise to a second and third pool of progenitors known as the basal progenitors (BP) and outer radial glia (oRG), respectively. These progenitors may divide either symmetrically producing either two identical daughter progenitors or two newborn neurons (indirect neurogenesis), thus amplifying the neuronal output from each AP [reviewed in Laguesse et al. (2015b)] (Figure 2, left). This transition from direct to indirect neurogenesis during cortical development occurred relatively recently in vertebrate evolution, and favored the increasing complexification of mammalian cerebral cortices when compared to other vertebrates (Dugas-Ford and Ragsdale, 2015). Laguesse et al. (2015b) first demonstrated that this gradual shift from direct to indirect neurogenesis is regulated by a progressive decrease in the UPR basal activity within AP, whereby its impairment leads to microcephaly. Taken together, these reports posit the following: (i) how does Zika induce microcephaly?; and more specifically (ii) does ZIKV perturb ER function or induce UPR response in ZIKV-infected fetuses?; (iii) does ZIKV unhinge the transition from direct to indirect neurogenesis?; (iv) what are the neurological sequelae of disrupted neurogenesis and corticogenesis in infants with congenital ZIKV? We launched a research program to answer these questions, and elucidate the molecular bases of ZIKV-associated microcephaly (Gladwyn-Ng et al., 2018).

## Zika Infection Deregulates UPR During Cortical Neurogenesis

We first performed transcriptomic and histological analyses of ZIKV-infected human fetal microcephalic cortices (median age of 22 gestational weeks), and uncovered a distinct up-regulation of UPR effectors. These data were further confirmed by the analysis of transcripts from both induced human neural progenitor cells (hNPCs) and mouse cortical cells that had been experimentally infected with ZIKV. Interestingly both PERK and IRE-1α downstream effectors were also strongly up-regulated. By using intraplacental (IPL) and intracerebroventricular (ICV) infections in combination with *in utero* electroporation (IUE), we were able to model ZIKV infection in wild-type embryonic outbred mice (Tuttle et al., 2018). These methodologies allowed further interrogation of the putative links between ZIKV infection, ZIKV-induced defects of corticogenesis and neurogenesis, as well as UPR deregulation (Gladwyn-Ng et al., 2018). Firstly, we observed ZIKV-infected embryonic cortices to be microcephalic (reduced both in tangential and radial dimensions), with disorganized cortical layering, as well as the tropism of ZIKV for neuronal progenitors and neurons, all of which are consistent with previous studies (Cugola et al., 2016; Li et al., 2016; Wu et al., 2016). We further demonstrated that neuronal progenitors within ZIKV-infected brains exhibited ER stress markers (e.g., upregulation of Calreticulin and Calnexin), and were disinclined to transit from direct to indirect neurogenesis prior to massive apoptosis of their progeny daughter cells (projection neurons) (Figure 2, right). These findings are supported by subsequent reports of transcriptomics analyses of ZIKV-infected cells that revealed a distinct up-regulation of UPR effectors (Rolfe et al., 2016; Kozak et al., 2017; Gurumayum et al., 2018). These conclusions were later supported by multiple investigations of the ZIKV in immunodeficient mice (Garcez et al., 2018; Kum et al., 2018), such as the intraperitoneal injection of ZIKV into mice lacking Type I and II IFN receptors that subsequently exhibited significant up-regulation of the expression of key markers of ER stress in the neural cells (Tan et al., 2018). To investigate the observed Zika tropism for progenitors and neurons, we infected embryonic mouse brains with two other flaviviruses [West Nile and yellow fever (vaccine 17D strain) viruses]and demonstrated that defects in corticogenesis and neurogenesis were specific to ZIKV.

We next utilized GSK2656157 and 4μ8C, which are specific inhibitors of the PERK and IRE-1α, respectively, to investigate if these compounds could ameliorate ZIKV-induced UPR-mediated cortical defects. Of note, oral administration of GSK2656157 has been reported to cross the blood-brain barrier and possess preventive properties against tau-mediated neurodegeneration in a mouse model of frontotemporal dementia (Radford et al., 2015) and clinical disease in prion-infected mice (Moreno et al., 2013), while 4μ8C is undergoing preclinical trials in the treatment of multiple myeloma (Cross et al., 2012). We were able to significantly rescue cortical dimensions, neurogenic defects by modulating cortical progenitors’ specification as well as to prevent the excessive cell death observed in infected mouse brains that were co-administered with either of these UPR inhibitors. Moreover, the genetic suppression of C/EBP homologs protein (Chop) (a pro-apoptotic factor induced by protracted UPR up-regulation, and a common effector gene of PERK and IRE-1α) by co-in utero electroporation of a specific siRNA reduced the apoptotic events within ZIKV-infected mouse cerebral cortices. These findings also directly implicated the terminal UPR pathway in the cell death observed in ZIKV-infected brains in addition to compromised direct neurogenesis.

## ZIKV Induces ER Stress and Cell Death Via Cell-Autonomous and Non-Cell-Autonomous Ways

The disrupted neurogenesis and elevated cell death of positively and negatively ZIKV-immunolabelled cells within infected brains could be rescued by co-administration of the above-stated UPR inhibitors. These findings further indicated that ZIKV also alters cortical cell behavior and survival in a non-cell-autonomous way, which have been further substantiated in other reports (Olmo et al., 2017), and ZIKV might induce UPR deregulation in a direct and indirect manner. This suggests that ZIKV-infected cells may release signaling molecules that influence UPR activity of neighboring cells, either as an anti-viral defense mechanism or to favor ZIKV replication. Correspondingly, ZIKV infection was recently reported to induce a strong release of glutamate and cytokines (e.g., IL-8, IL-1ß, IL-6, CXCL10, and TNF-alpha) that have a cytotoxic effect on both infected and uninfected neurons. Specifically, high doses of interleukins are known to induce ER stress and cell death (Zhang and Kaufman, 2008; Smith, 2018), while ZIKV induces chemokine CXCL10 that stimulates the NMDA receptor (NMDAR) (Ye et al., 2013; Li et al., 2016; Olmo et al., 2017) and exacerbates NMDAR-mediated cytotoxicity through activation of the interferon-gamma (IFN-γ) regulated pathways (van Weering et al., 2011; Chaudhary et al., 2017). The combinatorial release of interleukins and chemokines also possesses the indirect ability to attract and activate microglia, the resident immune cells in the CNS, to the site of infection (Hanisch, 2002). The activation of microglia upon ZIKV infection, via the cell-surface receptor tyrosine kinase (encoded by the AXL gene) (Lum et al., 2017; Meertens et al., 2017; Diop et al., 2018; Wang et al., 2018), may be due to a direct up-regulation of the ATF6 pathway (Ta et al., 2016). ZIKV induces the secretion of chemokines and other cytotoxic molecules from infected microglia by activating both UPR and toll-like receptor 3 (TLR3) signaling (Hosoi et al., 2013; Kim et al., 2015; Wen et al., 2017). Hence, the infection of microglia by ZIKV may promote viral replication by inducing cell cycle arrest and cell differentiation (Wang et al., 2018). In addition, microglia is a potential reservoir of viruses in the CNS (biorxiv, https://doi.org/10.1101/142497) and, by releasing TNF-α and other molecules, microglia induces the apoptosis of neural precursors (Ghosh and Basu, 2009; Guadagno et al., 2013). Furthermore, ZIKV can infect astrocytes during perinatal stages and adulthood (Limonta et al., 2018; Stefanik et al., 2018) and probably induces the production of several chemokines and cytokines mainly through UPR activation, since IFN-modulated pathways regulating cytokines production, such as the Jak1/Stat3 signaling, are efficiently inhibited by Zika infection (O’Shea and Plenge, 2012; Cao et al., 2017). Microglia, in turn, releases IL-6 cytokines, which synergize with ER stress within astrocytes, to further promote inflammatory responses (Meares et al., 2014). This feed-forward loop is most probably at the basis of CD8+ T cell infiltration in CNS, since high levels of IL-6 and CXCL10 release and ER stress induction in astrocytes might be the main cause of breakdown of the brain blood barrier and lymphocyte chemoattraction/activation (Lunardi et al., 2015; Lee et al., 2016; Manangeeswaran et al., 2016; Jurado et al., 2018). Upon infiltration of the immune privileged CNS and/or PNS (e.g., the dorsal root ganglion) by these activated CD8+ T cells and other lymphocytes, their cellular activities may contribute to the neurological sequelae of perinatal and adult ZIKV infection (Manangeeswaran et al., 2016; Huang et al., 2017; Zukor et al., 2018). Interestingly, interleukin-induced UPR activation might underlie lymphocyte-mediated neuronal damages since this pathway also has important roles in the activation of the adaptive immune pathway (Kamimura and Bevan, 2008; Grootjans et al., 2016). Thus, direct and/or indirect induction of UPR responses by ZIKV may regulate both innate and adaptive immune responses (Smith, 2014; Sprenkle et al., 2017) and be crucial for the insurgence of ZIKV pathogenesis and co-morbidities.

Further exploration of this signaling system by delineating the temporal dynamics between viral infection, replication, and dissemination with the co-administration of UPR inhibitors would be useful toward elucidating the relationships between UPR, cell proliferation and death to construct a more exhaustive understanding of interactions of flaviviruses with host cells and uncovering novel therapeutic targets.

## Perspectives

Our work has improved the understanding of ZIKV-induced defects by providing a more comprehensive perspective to other molecular studies that implicate different pathways in the cellular response to ZIKV infection. For example, the observed up-regulation of the pro-apoptotic factor p53 in PC12 cells overexpressing different ZIKV structural proteins and the consequent activation of a caspase-9- and caspase-3-mediated apoptotic pattern (Liu et al., 2018) might be attributable to a consistent and prolonged UPR activity. Indeed, Lin et al. (2012) demonstrated that UPR regulates p53 expression through modulation of NF-kB, which in turn, has a crucial role in several processes such as cell proliferation, differentiation, apoptosis, and inflammation and immune responses. Another report demonstrated that ZIKV acts, at least in part, by inhibiting the Akt-mTOR pathway, hence inducing strong autophagy and a block of neurogenesis (Liang et al., 2017). Previous work clearly showed that UPR up-regulation modulates autophagy through the same pathway (Qin et al., 2010). Finally, it was recently proposed that a ZIKV-induced increase in p53 expression and a deregulation of the mTOR pathway might induce early shifting from glycolysis to oxidative phosphorylation (OXPHOS) in mature myelin-producing cells notably in Schwann cells of peripheral nervous system. These recent reports suggest that activation of p53 is an early, albeit specific event in ZIKV infection that may result from cell- or non-cell- autonomous mechanisms (Devhare et al., 2017; Liu et al., 2018). These cellular events could also unsettle the homeostatic demands on cell proliferation, differentiation and metabolism, and potentially perturb cellular activities that lead to neurological diseases such as Guillain-Barre syndrome (Rothan et al., 2018). Thus, ZIKV-induced UPR deregulation may account for many of the defects observed in infected brains such as brain calcification, cell cycle and neurogenic impairments, cell death increase and cortical laminar defects.

Whilst clinicians and epidemiologists continue to investigate the distribution of ZIKV amongst different human populations in order to identify potential biological and socio-economic co-acting risk factors (Campos et al., 2015), clinical case studies remain sparse in comparison to molecular studies in animal and cellular models *in vivo* and *in vitro*, respectively. Recent gynecological reports demonstrate that the dramatic presentation of microcephaly in ZIKV-infected fetuses is not the most prominent clinical presentation, and is overshadowed by radiological findings of ventriculomegaly, cortical atrophy, calcifications (particularly located at the cortico-subcortical junction), and anomalies of the corpus callosum (Netto et al., 2017; Schaub et al., 2017a). Furthermore, emerging pediatric case reports of congenitally ZIKV-infected infants support that the spectrum of fetal brain and other anomalies is broader and more complex than microcephaly alone and includes subtle fetal brain injuries (Walker et al., 2018), ocular and auditory defects (Peloggia et al., 2018), as well as histopathological findings of viral dissemination and detection within extra-CNS tissues of post-mortem samples (Valdespino-Vazquez et al., 2018).

## Conclusion

With the global increase in arthropod- and mosquito-borne diseases, it remains imperative to better understand the epidemiology and pathophysiology underlying the teratogenic effects ZIKV and other related arboviruses [reviewed in Charlier et al. (2017)]. The combined efforts of the public with clinical, translational, and fundamental research will hopefully help limit the health and economic burden associated with emerging arboviruses and help limit their associated morbidity.

## Author Contributions

CA and IG-N wrote the manuscript with the help of the other authors. All authors edited the manuscript.

## Conflict of Interest Statement

The authors declare that the research was conducted in the absence of any commercial or financial relationships that could be construed as a potential conflict of interest.

## Footnotes

1 http://www.who.int/mediacentre/news/statements/2016/1st-emergency-committee-zika/en/
